# Efficacy of exercise-based interventions for pain intensity in children and adolescents with nonspecific chronic low back pain: a systematic review with meta-analysis

**DOI:** 10.3389/fphys.2026.1729972

**Published:** 2026-03-26

**Authors:** Zijing Jiang, Ying Hou, Yuliang Sun

**Affiliations:** School of Physical Education, Shaanxi Normal University, Xi’an, China

**Keywords:** back exercise program, home exercise, low back pain, meta, analysis, truck stabilization exercise

## Abstract

**Background:**

Exercise interventions represent a widespread approach for managing nonspecific chronic low back pain (CLBP) in pediatric and adolescent populations, either as standalone treatments or in combination with supplementary therapies. However, the magnitude of their impact on pain severity has not been systematically quantified.

**Objective:**

To meta-analyze the effects of (1) standalone exercise therapy *versus* no intervention/usual care and (2) exercise therapy plus adjunctive therapies *versus* exercise therapy alone on pain intensity in children and adolescents with nonspecific CLBP.

**Data sources:**

Five databases (Cochrane Library, Medline, Web of Science, PubMed, Embase) were searched from inception to January 2026, restricting included studies to those published in English.

**Study selection:**

nonspecific CLBP patients aged 6–19 years were selected. Inclusion criteria mandated that studies be controlled clinical trials featuring pre-intervention and post-intervention assessments, with reported measures of pain intensity.

**Data Extraction:**

Two reviewers independently executed data extraction and bias risk evaluation.

**Result:**

In a meta-analysis of two trials (*n* = 125), exercise-based interventions compared with no intervention/usual care showed an uncertain effect on pain intensity (SMD = −0.99, 95% CI −9.00 to 7.01), with substantial heterogeneity. Interventions combining exercise with adjunctive therapies were associated with a small additional reduction in pain intensity compared with exercise alone (3 studies, *n* = 280; SMD = −0.38, 95% CI −0.56 to −0.20).

**Conclusion:**

Evidence from randomized trials is limited, and the overall certainty is very low. Exercise-based interventions may reduce pain intensity compared with no intervention/usual care, but the estimate is highly uncertain because of very serious imprecision. Adding adjunctive therapies to exercise may provide a small incremental reduction in pain, although confidence in this effect remains low, and further well-designed, adequately powered trials are needed.

## 1 Introduction

Low back pain (LBP) is the most common type of back pain, the leading cause of disability, and the most common of all non-communicable diseases (Owen et al., 2020). Chronic low back pain (CLBP) is defined as pain localized between the inferior gluteal folds and the costal margin, with or without leg pain, persisting for at least 12 weeks (Koes et al., 2010). In clinical settings, up to 90% of CLBP cases cannot be linked to a specific pathology; hence, these individuals are categorized as having nonspecific CLBP (Maher et al., 2017). Among children and adolescents, epidemiological studies show that the incidence of lower back pain is showing a worrying upward trend, and the rate is close to that of adults (Balagué et al., 1999; Ebbehøj et al., 2002). A systematic review by Vidal-Conti et al. (2021) reported a pooled point prevalence of 84% in children aged 10–12 years. In terms of the overall incidence, it has been shown that the accumulated lifetime prevalence rate is 30%–51% according to subjective feeling; It is between 14% and 43% under objective observation (Balagué et al., 1999). The average annual incidence of LBP in this population is estimated to be about 16%; among them, 50% are recurrent, and about 8% progress to a chronic condition (Balagué et al., 1999; Airaksinen et al., 2006). Therefore, these epidemiological characteristics indicate that a significant number of children with low back pain are not recovering spontaneously. As a common childhood complaint, if it shifts to a recurrent or chronic condition, it may not only highlight the potential for long-lasting functional impairment but also require targeted early intervention strategies specifically designed for younger patients urgently.

The nonspecific CLBP in children and adolescents can be better understood within a biopsychosocial framework. Biologically, based on the research results, children and adolescents with nonspecific lower back pain have severe deficiencies in hip joint mobility, spinal flexibility, and trunk muscle endurance (Noll et al., 2022; Sjolie, 2004). These biological defects can reduce the stability of the spine, thereby increasing the risk of chronicity and recurrence (Jones et al., 2005). Beyond peripheral musculoskeletal dysfunction, persistent pain during childhood and adolescence may also interfere with ongoing neurodevelopmental processes. Neuroimaging studies have shown that children and adolescents with chronic pain exhibit changes in brain structure and resting-state functional connectivity within key pain-related networks, including the default mode and emotion regulation networks (Bhatt et al., 2020). As can be seen from these results, in nonspecific CLBP patients, the biological deficiencies have extended from peripheral biomechanical defects to central nervous system damage, and prolonged pain has occurred throughout a critical period of growth. At the same time, neuromuscular control and trunk stability are still in the development stage in childhood and adolescence, which may affect both motor control strategies and pain response (Lopes et al., 2020). Under this development, the common recommendation of exercise or rehabilitation may also be effective depending on whether people can maintain it over time (Holt et al., 2020).

Psychologically, a meta-analysis by Beynon et al. (2020) found that psychological distress and emotional coping difficulties, such as depression and anxiety symptoms, as the most likely risk factors for back pain in children, adolescents, and young adults. Notably, adolescence marks a pivotal phase for the maturation of affect regulation and executive control functions, and chronic pain may interfere with these processes by continuously activating stress-related and threat-related neural systems. Longitudinal evidence shows that young people with chronic pain are at higher risk of developing adverse mental health issues in later life, including an increased likelihood of psychiatric disorders (Murray et al., 2020). These findings underscore that chronic pain is not solely a transient sensory phenomenon but rather a psychological stressor capable of reshaping emotional development and pain-related cognitions, potentially fostering maladaptive pain behaviors over time.

In terms of society, current research shows that nonspecific chronic low back pain in children and adolescents not only has physical symptoms but is also accompanied by considerable social behavioral problems. First of all, those affected have a significantly higher rate of school absence; the more severe the pain, the more frequent the absence (Groenewald et al., 2019). At the same time, patients often exhibit avoidance behaviors towards physical activity and have lower participation in sports. This decrease also weakens their physical condition and the frequency of contact with peers, thereby further exacerbating psychological problems and social skills impairment (Agoston et al., 2016; Jones et al., 2004). Collectively, these social disruptions may enhance the functional limitations and interact with biological and psychological factors to form a cycle of pain and reduced participation.

In summary, the biological, psychological, and social factors outlined above offer a broad contextual framework for comprehending nonspecific CLBP in children and adolescents. This perspective emphasizes the multidimensional nature of the condition and the intricate interactions that shape pain persistence and functional outcomes during critical developmental periods.

Despite this significant burden, the management of nonspecific CLBP in pediatric populations remains challenging and is notably limited compared to adults. Invasive and pharmacological interventions, which are sometimes used in severe adult cases (Airaksinen et al., 2006), are rarely appropriate for children and adolescents due to developmental considerations, high costs (Hong et al., 2013), and potential risks (Frosch et al., 2022). As a result, clinical management in this age group depends almost entirely on conservative measures (Kosseim et al., 2008). Under such circumstances, therapeutic exercise, as a foundational intervention, can directly address modifiable biomechanical deficits in order to foster the development of healthy movement patterns and self-management skills during a critical developmental window (Leite et al., 2022). It also provides a favorable balance between therapeutic benefit and cost-effectiveness (García-Moreno et al., 2025; Zaina et al., 2025). Furthermore, it is worth noting that regardless of the delivery setting (supervised or home-based), strategies to enhance patient adherence are considered crucial for sustaining the benefits of exercise therapy over time (Michaleff et al., 2014).

However, despite its strong theoretical rationale, definitive evidence regarding exercise therapy’s effectiveness in reducing pain intensity within this specific population remains scarce. A recent network meta-analysis by García-Moreno and colleagues (García-Moreno et al., 2025) provided a wide-ranging overview of exercise interventions for nonspecific low back pain in this age group; however, it incorporated both acute and chronic conditions. Given the distinct characteristics of chronic pain, a synthesis focused exclusively on CLBP is necessary to generate more precise, clinically applicable estimates of exercise’s effect on pain intensity—both when used alone and when combined with other therapeutic approaches. Therefore, by synthesizing evidence from randomized controlled trials (RCTs), this study seeks to determine and critically evaluate the effectiveness of exercise therapy, both as a standalone intervention and when paired with additional treatments, in reducing chronic low back pain among this population.

## 2 Materials and methods

This review and meta-analysis were conducted in accordance with the PRISMA 2020 guidelines (Page et al., 2021). Prior to initiating literature retrieval, it was prospectively registered on PROSPERO (CRD420261307430).

### 2.1 Data sources and searches

A thorough literature search explored the association between exercise treatment and low back pain in children and adolescents. The literature search was conducted by Z.J. across the Cochrane Library, MEDLINE, Web of Science, PubMed, and Embase, with studies being searched from the time of build to 14 January 2026. Controlled terms such as MeSH for PubMed and Emtree for Embase, along with relevant free-text keywords including “exercise,” “training,” “adolescents,” “child,” “low back pain,” and “chronic low back pain,” were used to construct a structured search strategy. Boolean operators (AND, OR) were applied to logically combine synonyms within each conceptual group (using OR) and to link the different groups together (using AND). To ensure a comprehensive retrieval of potentially eligible studies and mitigate potential publication bias, the search strategy was augmented by executing both backward and forward citation tracking (Hirt et al., 2023). Duplicate records were first eliminated with EndNote (version 21; Clarivate, Philadelphia, PA, United States of America). The screening process then proceeded in two phases. First, records were screened by title and abstract to assess their relevance based on predefined criteria, including subject area, study design, population, and disease conditions. Subsequently, full-text articles of potentially eligible studies underwent thorough review for more detailed evaluation against specific inclusion and exclusion criteria, such as pain duration, types of interventions, and outcome measures. To establish preliminary eligibility, two authors (Z. J. and Y. S.) independently screened the titles and abstracts, after which they evaluated the full texts. Whenever disagreements arose, they were discussed; if consensus could not be reached, a third reviewer (Y.H.) provided a final decision. The complete search strategy is provided in Supplementary Appendix S1.

### 2.2 Eligibility criteria

Inclusion criteria: (1) The study design is a randomized controlled trial (RCT); (2) Participants must be children and adolescents aged 6–19 years; (3) Pain duration ≥12 weeks; (4) There should be no significant difference between the experimental group and the control group before exercise intervention; (5) Experimental intervention involves any physical exercise. Exclusion criteria: (1) Adults with comorbidities or over 19 years old; (2) The pain duration <12 weeks; (3) Intervention methods excluding exercise therapy; (4) The studies in Chinese and other languages will not be considered for inclusion. Our criteria for including studies obeyed the PICOS principle: P (Population) children and adolescents aged 6–19 years with nonspecific chronic low back pain (duration ≥3 months); I (Intervention) any structured exercise-based intervention; C (Comparison) (1) exercise-based interventions *versus* no intervention/usual care control, and (2) exercise plus adjunctive therapy *versus* exercise alone; O (Outcome) quantitative pain intensity outcomes reported as mean and SD; S (Study design) peer-reviewed randomized controlled trials.

### 2.3 Data extraction

All variables were dually extracted by independent reviewers (Z.J. and Y.S.) for each investigation. The extracted data was arbitrated by the third reviewer (Y.H.) to settle disagreements between the two reviewers. The extracted information from the study encompasses multiple dimensions, such as research characteristics (publication year, country, first author, and experimental design), participant characteristics (age, sample size, gender distribution, pain duration and baseline pain level), intervention specifics (intervention strategies, intervention time, frequency and duration of training sessions), as well as outcome measures. Two reviewers independently screened the studies to eliminate irrelevant ones, with any disagreements addressed through collective discussion. When information was incomplete, we contacted the original authors to request missing data. The process of data selection and extraction was carried out between 14 January 2026, and 1 February 2026.

### 2.4 Literature quality assessment

The risk of bias was assessed using the Cochrane Risk of Bias 2 (RoB 2) tool, which evaluates bias across five domains: randomization process, deviations from intended interventions, missing outcome data, measurement of the outcome, and selection of the reported result. Two authors (Z.J. and Y.S.) independently conducted the assessments following the guidance provided in the Cochrane RoB 2 tool manual and the accompanying Excel tool (Sterne et al., 2019). Each domain received a rating of low risk, some concerns, or high risk, depending on the assessment. The overall RoB was determined by the worst judgment across all domains. In cases where multiple domains were judged as “some concern,” the overall classification was “high RoB,” consistent with the guidelines provided by the tool’s authors.

### 2.5 Data synthesis and statistical analysis

All models were performed using R software (version 4.5.2, R Studio) using the *metafor* package (Viechtbauer, 2010), and later visualized with the *ggplot2* package (Rodríguez et al., n.d.). As all outcome data in the study were continuous variables, the analyses were conducted using standardized mean difference (SMD) with 95 percent confidence intervals (95%CI) as the measures of effect size. For the comparison of exercise-based interventions *versus* no intervention/usual care, we extracted pain intensity data from the intervention and control groups and pooled between-group SMDs based on post-intervention values. For the comparison of exercise plus adjunctive therapy *versus* exercise alone, we likewise pooled between-group SMDs using post-intervention values. Since both groups received the same basic exercise therapy, the differences between the two groups after the intervention could be more reliably attributed to the additional effect of the supplementary therapy. Heterogeneity in the study was evaluated using the *I* ^2^ statistic. A random effects model was applied for all meta-analyses due to anticipated clinical and methodological diversity. To account for uncertainty in the estimation of between-study variance, especially given the small number of studies, we used the Hartung-Knapp-Sidik-Jonkman (HKSJ) adjustment for calculating confidence intervals around the pooled effect estimates. This method provides more conservative and reliable intervals when the number of studies is limited (IntHout et al., 2014). Statistical significance was defined as a p-value less than 0.05. Because each meta-analysis included fewer than 10 studies, formal tests for funnel plot asymmetry (e.g., Egger’s regression) were not considered reliable and were therefore not used to draw conclusions about publication bias. Funnel plots, when presented, are descriptive only.

### 2.6 The overall strength of the evidence

Two authors (Y.H. and Z.J.) evaluated the certainty of evidence for the outcomes using the Grading of Recommendations Assessment, Development and Evaluation (GRADE) approach *via* the GRADEpro Guideline Development Tool (Higgins et al., 2011). The evaluations were categorized into five domains: risk of bias, inconsistency, indirectness, imprecision, and publication bias. Each domain was assessed based on the evaluation criteria, and evidence was downgraded where applicable (Table 1).

**TABLE 1:** GRADE Summary of Findings: Pain intensity (SMD). Negative SMD indicates lower pain intensity and favors the intervention.

Outcome	Comparison	Studies	Participants	SMD	95% CI	Certainty
Pain intensity (SMD)	Exercise + adjunct vs. exercise	3	280	−0.38	−0.56 to −0.20	⊕◯◯◯ very low
Pain intensity (SMD)	Exercise vs. No intervention/Usual care	2	125	−0.99	−9.00 to 7.01	⊕◯◯◯ very low

Outcome	Comparison	RoB	Inconsistency	Indirectness	Imprecision	Pub bias
Pain intensity (SMD)	Exercise + adjunct vs. exercise	Serious^a^	Not serious	Not serious	Serious^b^	Not assessed^c^
Pain intensity (SMD)	Exercise vs. No intervention/Usual care	Serious^a^	Serious^d^	Not serious	Very serious^e^	Not assessed^c^

Abbreviations: CI, confidence interval; GRADE, grading of recommendations assessment, Development and Evaluation; Pub bias, publication bias; RoB, risk of bias; SMD, standardized mean difference.

^a^ Downgraded one level for risk of bias (overall RoB2 concerns judged as serious across included trials).

^b^ Downgraded one level for imprecision because the CI, includes both meaningful benefit and no effect (crosses the null).

^c^ Publication bias was not assessed because fewer than 10 studies were available.

^d^ Downgraded one level for inconsistency due to substantial heterogeneity/variation in effect estimates across studies.

^e^ Downgraded two levels for imprecision due to an extremely wide CI, spanning large benefit and large harm, indicating very serious uncertainty.

## 3 Results

### 3.1 Literature search results

**FIGURE 1:**
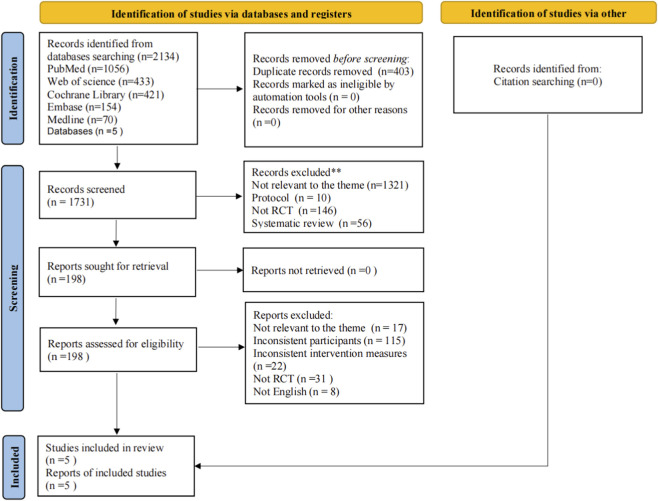
Flow diagram of study selection.

The study selection procedure is summarized in the relevant flowchart, along with the justifications for excluding specific studies. A total of 2,134 records were identified through database searches using available search terms and synonyms from inception to 10 January 2026. A total of 1,731 studies remained after eliminating 403 duplicate studies. Following title and abstract review, 1,533 papers were eliminated. After a comprehensive full-text analysis of the remaining 198 papers, 193 were disqualified in accordance with the established inclusion and exclusion standards. During full-text screening, most studies were excluded due to ineligible study populations, non-exercise-based interventions, pain duration shorter than 12 weeks, or the absence of quantitative pain intensity outcomes required for meta-analysis. The final sample includes five studies (Jung et al., 2020; Evans et al., 2018; Ahlqwist et al., 2008; Fanucchi et al., 2009; Jones et al., 2007). The article screening process is shown in Figure 1.

### 3.2 Basic information on the inclusion of the literature

The effects of exercise treatment on pain intensity in children and adolescents with nonspecific CLBP were investigated in 5 articles with 405 participants. The five included studies were categorized into two comparison groups based on their intervention designs. The first group, Exercise Therapy vs. no intervention/usual care (2 studies) (Jones et al., 2007; Fanucchi et al., 2009), compared structured exercise programs with no intervention/usual care control. The second group, Combined Exercise Therapy vs. Exercise Alone (3 studies) (Ahlqwist et al., 2008; Evans et al., 2018; Jung et al., 2020), evaluated the additional benefit of adjunctive therapies (e.g., spinal manipulative therapy, vibratory stimulation, individualized physical therapy) when combined with exercise compared to exercise alone. The outcome indicators were all pain score scales. Two studies used the Numerical Pain Rating Scale (NPRS) (Evans et al., 2018; Jung et al., 2020), two studies used the Visual Analogue Scale (VAS) (Ahlqwist et al., 2008; Fanucchi et al., 2009), and one study used an unspecified 0–10 pain scale (Jones et al., 2007). The research characteristics are shown in Table 2. To ensure transparent reporting of the exercise interventions, the characteristics of each intervention are detailed in Table 3 using the Consensus on Exercise Reporting Template (CERT) (Slade et al., 2016) as a guide.

**TABLE 2:** Basic characteristics of included studies.

Research	Study design	Region	Sample size	Age (mean ± SD)	Sex (male/female)	Pain duration	Baseline pain level (mean)
Evans et al. (2018)	Parallel	USA	185	15.3 ± 1.8	58/127	≥12	>5
Jung et al. (2020)	Parallel	Korea	50	18.0 ± 0.65	28/22	≥12	>4
Ahlqwist et al. (2008)	Parallel	Sweden	45	15.0 ± 1.25	14/31	≥12	>4
Fanucchi et al. (2009)	Parallel	South Africa	71	12.0 ± 0.7	32/39	≥12	>4
Jones et al. (2007)	Parallel	UK	54	14.6 ± 0.6	-	≥12	>5

Research	Experimental group	Control group	Intervention course (week)	Outcome indicators	Training frequency	Duration of training (minute)
Evans et al. (2018)	Manipulative therapy + home exercise	Home exercise	52	NPRS	2/week	20–40
Jung et al. (2020)	Vibratory stimulation + trunk stabilization exercise	Trunk stabilization exercise	12	NPRS	3/week	25
Ahlqwist et al. (2008)	Individualized physical therapy + back exercise program	Back exercise program	12	VAS	E: 2/week C: 3/week	20
Fanucchi et al. (2009)	Exercise program and weekly home exercise program	No intervention	12	VAS	3/week	-
Jones et al. (2007)	Exercise rehabilitation program	Usual care	8	Unspecified 0–10 scales	2/week	30

NPRS, numerical pain rating scale; VAS, visual analogue scale; -, unspecified; C, control group; E, experimental group.

**TABLE 3:** Consensus on exercise reporting template.

Study, year Item	Evans et al. (2018)	Jung et al. (2020)	Ahlqwist et al. (2008)	Fanucchi et al. (2009)	Jones et al. (2007)
1. Equipment	0	1	0	0	0
2. Instructor qualifications	1	1	1	1	0
3. Individual or group	1	0	0	1	0
4. Supervision	0	1	1	0	0
5. Adherence description (measured and reported)	1	0	0	0	1
6. Motivation strategies	1	0	0	0	0
7a. Rules for progression	0	0	1	0	0
7b. Description of progression	0	0	1	0	0
8. Exercise description	1	1	0	1	0
9. Home-exercise components	1	0	1	1	0
10. Description of nonexercised components	1	0	1	1	1
11. Adverse events	1	1	0	1	1
12. Setting described	1	0	0	1	1
13. Description of exercise interventions	1	1	1	1	1
14a. Generic or tailored exercises	1	1	1	0	1
14b. Description of tailored exercises	1	0	1	0	0
15. Level of exercises(beginner, intermediate, advanced)	0	0	1	0	0
16a. Measure of exercise adherence	1	0	0	1	1
16b. Reporting of adherence	1	0	0	1	1
Total score	14	7	10	10	8

### 3.3 Results of literature quality evaluation

**FIGURE 2:**
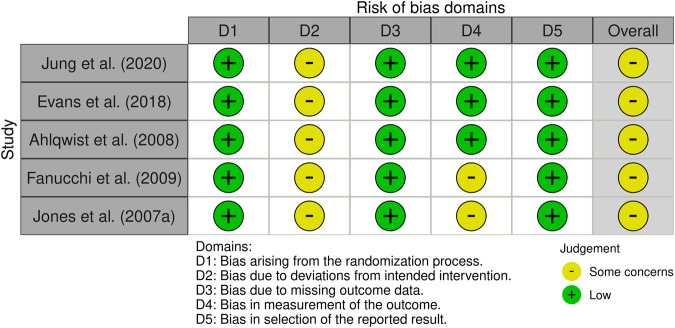
Diagram of the included literature with quality assessment.

**FIGURE 3:**
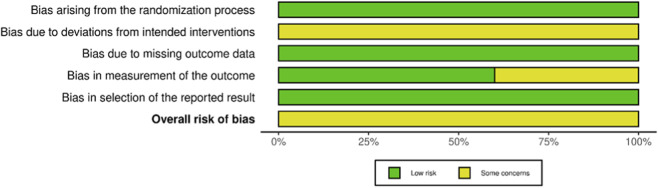
Summary diagram of the included literature with quality assessment.

The quality of all included studies (*n* = 5) was assessed using the Cochrane Collaboration (RoB 2) approach. For the randomization process (D1), all five studies described appropriate methods and were judged low risk. Regarding deviations from intended interventions (D2), all five studies raised some concerns. For missing outcome data (D3), all five studies were low risk. For measurement of the outcome (D4), four studies were low risk, while one study raised some concerns (Evans et al., 2018). For the selection of the reported result (D5), all five studies were of low risk. Overall risk of bias was rated as some concerns in all five studies; none was rated high risk. Detailed domain-level judgments are presented in Figures 2, 3.

### 3.4 Meta-analysis results

#### 3.4.1 Analysis of the therapeutic effect of exercise

**FIGURE 4:**
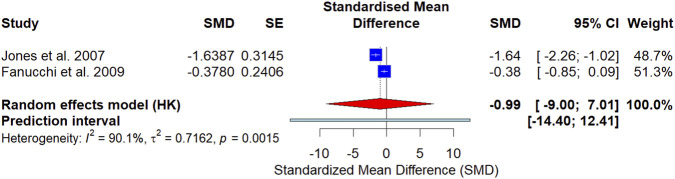
Forest plot of exercise therapy for posttest LBP intensity in nonspecific chronic low back pain.

Only two RCTs provided eligible data for the meta-analysis of standalone exercise therapy. Specifically, the exercise therapy analysis was conducted using between-group comparisons (exercise therapy vs. no intervention/usual care) from two RCTs (Jones et al., 2007; Fanucchi et al., 2009). The HKSJ-adjusted random-effects model showed that exercise therapy had an uncertain effect on nonspecific CLBP pain intensity in children and adolescents [SMD = −0.99, 95% CI (−9.00, 7.01), *p* > 0.05], as illustrated in Figure 4. A negative standardized mean difference (SMD) indicates lower post-intervention pain intensity in the exercise group compared with the control group, with lower scores representing better outcomes. The heterogeneity between studies was substantial (*I* ^2^ = 90.1%).

**FIGURE 5:**
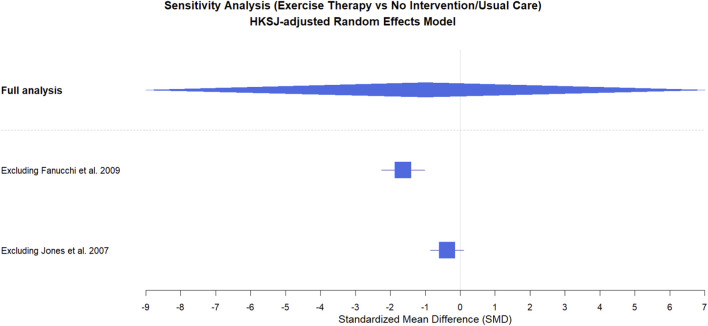
Sensitivity analysis of the effect of exercise therapy on nonspecific chronic low back pain.

To examine the robustness of the pooled estimate, we conducted a leave-one-out sensitivity analysis by sequentially excluding each study (Figure 5). Given that only two trials were available, the pooled effect was highly sensitive to the inclusion of individual studies, indicating limited robustness of the combined result. Publication bias was explored using a funnel plot; however, with only two studies, the plot is not informative and should be interpreted cautiously, and Egger’s test was not performed due to insufficient studies.

#### 3.4.2 Analysis of the effect of combined exercise therapy

**FIGURE 6:**
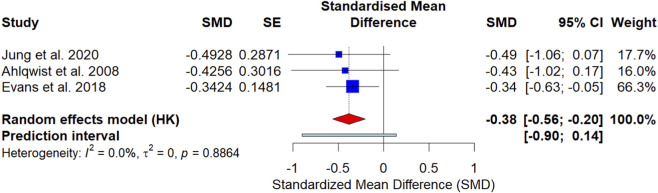
Forest plot of combined exercise therapy for posttest LBP intensity in nonspecific chronic low back pain.

This analysis evaluated the incremental benefit of adjunctive therapies added to exercise, based on between-group differences in post-intervention pain scores from three studies (*n* = 280) (Jung et al., 2020; Evans et al., 2018; Ahlqwist et al., 2008). The HKSJ-adjusted random-effects model demonstrated that combined exercise therapy showed a significant greater pain reduction compared to exercise therapy alone in nonspecific CLBP pain [SMD = −0.38, 95% CI (−0.56, −0.20), p < 0.05], as shown in Figure 6. A negative standardized mean difference (SMD) indicates that combined exercise therapy was more effective than exercise therapy alone in reducing pain intensity, as lower pain rating scale scores indicate a better outcome. The results showed that spinal manipulative therapy combined with exercise therapy [SMD = −0.34, 95% CI (−0.63, −0.05)], vibratory stimulation combined with trunk stabilization exercise [SMD = −0.49, 95% CI (−1.06, 0.07)], individualized physical therapy combined with exercise therapy [SMD = −0.43, 95% CI (−1.02, 0.17)]. Individual trial results were mixed, with two of the three studies showing confidence intervals that crossed the null, suggesting variability in effects across trials. The heterogeneity among the included studies was negligible (*I* ^2^ = 0.0%).

**FIGURE 7:**
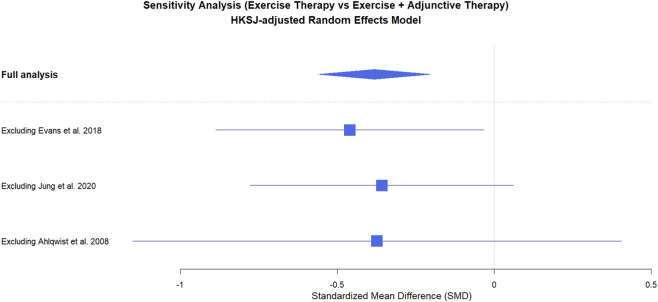
Sensitivity analysis of the effect of combined exercise therapy on nonspecific chronic low back pain.

Leave-one-out analyses showed that the pooled estimate remained negative across exclusions; however, confidence intervals widened and could include the null in some scenarios, indicating limited robustness due to the small number of studies (Figure 7). Because each meta-analysis included fewer than 10 studies, publication bias could not be reliably assessed. Therefore, funnel plots and Egger’s regression test were not performed (or, if presented, were used for descriptive purposes only), and no conclusions about the absence of publication bias were drawn.

## 4 Discussion

This meta-analysis synthesized randomized trial evidence on exercise-based interventions, alone or combined with adjunctive therapies, for reducing pain intensity in children and adolescents with nonspecific CLBP. The evidence base is limited and of very low certainty. Exercise-based interventions may reduce pain compared with a no-intervention/usual care, but the estimate is highly imprecise. Adding adjunctive therapies to exercise may provide a small additional benefit over exercise alone. Therefore, exercise-based interventions can be considered within an individualized, multimodal care plan, pending confirmation from future high-quality trials.

### 4.1 Exercise therapy

In children and adolescents aged 6–19 years with nonspecific chronic low back pain (CLBP), our pooled analysis of between-group post-intervention data suggested lower pain intensity following exercise-based interventions compared with no intervention/usual care control (SMD = −0.99, 95% CI −9.00 to 7.01). While the point estimate favors exercise and would be considered large by conventional benchmarks (Lakens, 2013), the extremely wide confidence interval spanning substantial benefit to no effect indicates very serious imprecision. Accordingly, the most defensible interpretation is not that exercise is definitively superior, but that current randomized evidence remains insufficient to quantify the comparative effect with confidence. This distinction is clinically important: it supports continued investigation and cautious use of exercise-based approaches, while avoiding overstatement of efficacy based on sparse data (Jones et al., 2007; Fanucchi et al., 2009).

The uncertainty of the pooled effect is consistent with both the small number of trials (*k* = 2) and substantial between-study variability (*I* ^2^ = 90.1%). Rather than being treated as a purely statistical artifact, this heterogeneity likely reflects meaningful clinical differences between the two included interventions. Jones et al. evaluate the structured Rehabilitation Program focusing on core stability and functional neuromuscular training; Fanucchi et al. delivered a program that combined structured physical education classes at school with home exercise guidance (Jones et al., 2007; Fanucchi et al., 2009). These approaches may differ in training specificity, progression, supervision, and adherence—features that can materially influence the achieved exercise “dose” and the contrast against the comparator condition. Notably, such variability is not limited to the included RCTs; Non-randomized adolescent studies have shown that trunk-focused exercise prescriptions can range from short-term core stability/stretching programs to long-term neuromuscular approaches, and the outcomes of these programs may differ accordingly (Arampatzis et al., 2021; Javadipour et al., 2021). Taken together, the available evidence suggests that the comparative effect of exercise may depend on the characteristics of the program and implementation, thereby explaining the current instability of a single pooled estimate.

Given the substantial heterogeneity and extreme imprecision, the pooled estimate should be interpreted cautiously; nonetheless, the direction of effect generally favored exercise over no intervention/usual care. This direction is consistent with exercise-focused trials in adolescents reporting improved pain outcomes, supporting plausibility but not establishing comparative efficacy (Selhorst and Selhorst, 2015; Dudoniene et al., 2016). Leite et al. (2022) proposed that exercise therapy is particularly salient in pediatric populations, as it targets modifiable biomechanical deficits and capitalizes on a critical developmental window to foster long-term functional resilience and healthy movement patterns. Future RCTs of pediatric exercise should clearly define comparators, report exercise prescriptions/adherence in detail, and harmonies outcome measures to determine the magnitude and duration of benefits.

### 4.2 Combined exercise therapy

The second treatment group consisted of children and adolescents aged 6 to 19 who had nonspecific chronic low-back pain (CLBP). Participants in this group received combined exercise therapy, which included the same structured exercise regimen as the control group, supplemented with adjunctive treatments such as manual therapy, vibratory stimulation and individualized physical therapy. Over three trials (*n* = 280), adding adjunctive therapies to exercise was associated with a small additional reduction in pain intensity compared with exercise alone (SMD = −0.38, 95% CI −0.56 to −0.20). However, certainty remains limited due to the small number of trials and variation in adjunctive components. Heterogeneity was negligible (*I* ^2^ = 0.0%), but given that there were few trials and different adjuncts, this should not be taken as evidence that various combination approaches are equivalent.

This pattern helps to contextualize mixed findings in the literature. For example, Parnell Prevost et al. (2019) reported additional benefit from manual therapy, whereas Dissing et al. (2018) observed no clear difference. Consistent with this, the pooled estimate indicates a small average incremental benefit of adjuncts added to exercise, which may contribute to the mixed results observed across individual trials, especially in smaller studies. Based on the above evidence, manual therapy can be regarded as one of the therapies combined with other methods; it should not be used alone as the main treatment (García-Moreno et al., 2025). Complementary signals are also observed in some individualized reports that integrate cognitive elements with movement training, as noted by Cañeiro et al. (2013), who described an improvement after using a cognitively guided functional method, and Ribas et al. (2022). Patients continue to improve in the program combining therapeutic exercise and motor imagery training. While such reports cannot establish comparative efficacy, they are directionally consistent with the possibility that some adolescents may benefit from augmenting exercise with cognitive/behavioral or imagery-based components.

Overall, combined exercise therapy may offer a modest additional benefit over exercise alone for pain intensity, but certainty remains limited and the optimal adjunct–patient match is unclear. Future pediatric RCTs should use clearly defined exercise-alone comparators, report exercise prescription/adherence in detail, and specify adjunct content and dose to determine when adjuncts meaningfully add to a well-designed exercise program.

### 4.3 Research limitations

There are several limitations to this meta-analysis. First, the number of eligible trials was small, limiting statistical power, widening uncertainty around pooled effects, and restricting exploration of heterogeneity (e.g., by exercise type, supervision, or dose). Second, intervention reporting was often incomplete (frequency, intensity, progression, adherence, and co-interventions), which weakens reproducibility and clinical interpretability of what “exercise therapy” entailed. Third, substantial between-study heterogeneity was observed in the exercise-versus-control comparison (*I* ^2^ = 90.1%), likely reflecting differences in exercise content, dose, comparator definitions (e.g., usual care/no intervention/physical activity), and outcome measurement; therefore, the pooled estimate should be interpreted cautiously and may not generalize across settings. Fourth, with few studies, publication-bias assessments (e.g., funnel plot and Egger’s test) were underpowered and cannot be considered definitive. Fifth, although random-effects models and the Hartung–Knapp–Sidik–Jonkman (HKSJ) method were used to better reflect uncertainty when few studies are available, these analytic choices cannot overcome limitations of the primary evidence (e.g., risk of bias and incomplete reporting). Finally, pain intensity was the main outcome and was not consistently accompanied by other patient-important endpoints (disability/function, quality of life, return to school/sport, adverse events, and longer follow-up).

### 4.4 Future applications

This meta-analysis indicates that exercise-based interventions may reduce pain intensity in adolescents with nonspecific CLBP; However, due to very low certainty of evidence and uncertainty about the magnitude of the benefit. Therefore, exercise may be regarded as a reasonable non-pharmacological option in individualized care, but current data are not sufficient to support strong claims that it is a first-line or basic treatment. For combined methods, adding auxiliary components to exercise may provide a minor additional benefit on average; however, this conclusion is drawn from a few trials and should not be taken as proof that different auxiliary strategies are equally effective or consistently so across various settings.

Looking forward, future studies should prioritize adequately powered pediatric RCTs with clearly defined comparators, and standardized outcome measurements and exercise prescriptions, as well as details on patient compliance, should be reported in greater depth. Especially need to explore the effect on the improvement of symptoms in a short period; more importantly, it is necessary to determine whether such benefits can be maintained for some time. Longitudinal follow-up is required to determine the durability of the effect, the practicability of implementation in routine care, and which patient subgroups may benefit most.

## 5 Conclusion

This meta-analysis provides very low certainty evidence that exercise therapy alone may reduce pain intensity in children and adolescents with nonspecific CLBP compared with no intervention/usual care, although the effect estimate is highly imprecise due to substantial heterogeneity. Combined exercise therapy with adjunctive treatments shows a small but statistically significant additional benefit over exercise alone, with low heterogeneity. Given the limited number of studies and methodological constraints, these findings should be interpreted cautiously. Further well-designed randomized controlled trials with larger sample sizes are needed to strengthen the evidence base and inform clinical practice guidelines.
