# Supplementary material for: Efficacy of exercise-based interventions for pain intensity in children and adolescents with nonspecific chronic low back pain: a systematic review with meta-analysis
Source: Front Physiol. 2026 Mar 26;17:1729972. doi: 10.3389/fphys.2026.1729972 (PMC13062903; doi:10.3389/fphys.2026.1729972)
Supplement: Supplementary file 1 [file Table1.docx]

Supplementary Material1

**1 The search strategy for PubMed 1056**

#1 MeSH Terms: adolescent

#2 Title/Abstract: adolescents OR childern OR child OR teenage OR youth OR pediatric

#3 MeSH Terms: Therapeutic Exercise OR Physical Therapy

#4 Title/Abstract: exercise OR sports OR training

#5 MeSH Terms: chronic low back pain OR non-specific low back pain

#6 Title/Abstract: low back pain OR non-specific low back pain OR chronic non-specific low back pain OR spinal pain

#7 #1 OR #2

#8 #3 OR #4

#9 #5 OR #6

#10 #7 AND #8 AND #9

**2 The search strategy for Web of Science 433**

#1 adolescent (Topic) or adolescents (Title) or childern (Title) or child (Title) or teenage (Title) and youth (Title) or pediatric (Abstract)

#2 Therapeutic Exercise (Title) or Physical Therapy (Title)

#3 exercise (Abstract) or sports (Abstract) or training (Abstract)

#4 #2 OR #3

#5 chronic low back pain (Title) or nonspecific low back pain (Title) or low back pain OR non-specific low back pain (Topic) or chronic nonspecific low back pain (Topic) or spinal pain (Topic)

#6 #1 AND #4 AND #5

**The search strategy for MEDLINE 70**

#6. #3 AND #4 AND #5….70

#5. low back pain (Title) or lower back pain (Topic) or nonspecific low back pain (Title) or non-specific low back pain (Topic) or chronic low back pain (Topic) or nonspecific chronic low back pain (Topic) or non-specific chronic low back pain (Topic) or lumbago (Topic) or lumbal pain (Topic) or lumbar pain (Topic) or lumbalgia (Topic) ….92,646

#4. adolescent (Title) or teenage (Title) or child (Title) or children (Title)….995,262

#3. #1 OR #2…..119,059

#2. corrective exercise (Topic) or exercise movement techniques (Topic) or exercise therapy (Author) or exercise treatment (Topic) or kinesiotherapeutic intervention (Topic) or kinesiotherapeutic method (Topic) or kinesiotherapeutic procedure (Topic) or kinesiotherapeutic technique (Topic) or kinesitherapeutic procedure (Topic) or kinesitherapeutical treatment (Topic)….118830

#1. kinesiotherapy (Topic) or kinesiotherapy (Topic)….310

**4 The search strategy for Cochrane Library**

#1 MeSH descriptor: [Adolescent] explode all trees 136093

#2 (Adolescents):ti,ab,kw 29506

#3 (Male Adolescents):ti,ab,kw 15298

#4 (Teen):ti,ab,kw 1064

#5 (Teenager):ti,ab,kw 128

#6 (Teenagers):ti,ab,kw 1272

#7 (Teens):ti,ab,kw 1347

#8 (Youth):ti,ab,kw 10478

#9 (Youths):ti,ab,kw 1367

#10 #1 OR #2 OR #3 OR #4 OR #5 OR #6 OR #7 OR #8 #9 154765

#11 MeSH descriptor: [Exercise Therapy] explode all trees 22445

#12 (exercise):ti,ab,kw 141188

#13 (Therapeutic exercise):ti,ab,kw 15674

#14 (physical therapy):ti,ab,kw 72085

#15 (sports):ti,ab,kw 10977

#16 (training):ti,ab,kw 140926

#17 #11 OR #12 OR #13 OR #14 OR #15 OR #16 278466

#18 MeSH descriptor: [Low Back Pain] explode all trees 6205

#19 ("chronic low-back pain"):ti,ab,kw 5270

#20 (nonspecific low back pain):ti,ab,kw 2621

#21 (chronic nonspecific low back pain):ti,ab,kw 1817

#22 (nonspecific chronic low back pain):ti,ab,kw 1817

#23 (spinal pain):ti,ab,kw 18118

#24 #18 OR #19 OR #20 OR #21 OR #22 OR #23 26277

#25 #10 AND #17 AND #24 421

**5 The search strategy for Embase**

Embase

Session Results

.......................................................

No. Query Results Results Date

#26. #10 AND #19 AND #24 154

#24. #20 OR #23 75,436

#23. ('back pain, low':ti,ab,kw OR 'chronic low back 22,614

pain':ti,ab,kw OR 'loin pain':ti,ab,kw OR 'low

backache':ti,ab,kw OR 'low backpain':ti,ab,kw OR

'lowback pain':ti,ab,kw OR 'lower back

pain':ti,ab,kw OR 'lumbago':ti,ab,kw OR 'lumbal

pain':ti,ab,kw OR 'lumbal syndrome':ti,ab,kw OR

'lumbalgesia':ti,ab,kw OR 'lumbalgia':ti,ab,kw OR

'lumbar pain':ti,ab,kw OR 'nonspecific low back

pain':ti,ab,kw OR 'chronic nonspecific low back

pain':ti,ab,kw) AND [embase]/lim

#20. 'low back pain'/exp AND [embase]/lim 72,284

#19. #14 OR #18 3,726,727

#18. #15 OR #17 3,198,679

#17. ('children':ti,ab,kw OR 'child':ti,ab,kw) AND 1,664,201

[embase]/lim

#15. ('child'/exp OR 'child') AND [embase]/lim 2,964,029

#14. #11 OR #13 1,401,247

#13. ('teenager':ti,ab,kw OR 'adolescent':ti,ab,kw) 191,653

AND [embase]/lim

#11. ('adolescent'/exp OR 'adolescent') AND 1,400,504

[embase]/lim

#10. #7 OR #9 89,036

#9. (('corrective exercise':ti,ab,kw OR 'exercise 3,139

movement techniques':ti,ab,kw OR 'exercise

therapy':ti,ab,kw OR 'exercise

treatment':ti,ab,kw OR 'kinesiotherapeutic

intervention':ti,ab,kw OR 'kinesiotherapeutic

method':ti,ab,kw OR 'kinesiotherapeutic

procedure':ti,ab,kw OR 'kinesiotherapeutic

technique':ti,ab,kw OR 'kinesiotherapeutical

treatment':ti,ab,kw OR 'kinesitherapeutic

exercises':ti,ab,kw OR 'kinesitherapeutic

intervention':ti,ab,kw OR 'kinesitherapeutic

method':ti,ab,kw OR 'kinesitherapeutic

methodology':ti,ab,kw OR 'kinesitherapeutic

procedure':ti,ab,kw OR 'kinesitherapeutic

technique':ti,ab,kw OR 'kinesitherapeutic

treatment':ti,ab,kw OR 'kinesitherapeutical

treatment':ti,ab,kw OR 'kinesitherapy':ti,ab,kw

OR sktm:ti,ab,kw) AND 'specialized

kinesitherapeutic methodology':ti,ab,kw OR

'specialised kinesitherapeutic

methodology':ti,ab,kw OR 'specialized

kinesitherapeutic methodology':ti,ab,kw OR

'therapeutic exercise':ti,ab,kw OR 'therapy,

exercise':ti,ab,kw OR 'treatment,

exercise':ti,ab,kw OR 'kinesiotherapy':ti,ab,kw)

AND [embase]/lim

#7. ('kinesiotherapy'/exp OR 'kinesiotherapy') AND 87,859

[embase]/lim

.......................................................
