# Supplementary material for: Efficacy of exercise-based interventions for pain intensity in children and adolescents with nonspecific chronic low back pain: a systematic review with meta-analysis
Source: Front Physiol. 2026 Mar 26;17:1729972. doi: 10.3389/fphys.2026.1729972 (PMC13062903; doi:10.3389/fphys.2026.1729972)
Supplement: Supplementary file 2 [file Table2.docx]

| **Table 1 Proforma CERT assessment form** | | | | | | |
| --- | --- | --- | --- | --- | --- | --- |
| **Author and year: Jung et al. 2020** | | | | | | |
| **Title: The Effectiveness of Trunk Stabilization Exercise Combined with Vibration for Adolescent Patients with Nonspecific Low Back Pain** | | | | | | |
| **Journal: International Journal of Environmental Research and Public Health** | | | | | | |
| **Study location: Korea** | | | | | | |
| **Reviewer and date** | | | | | | |
| **Item** | **Description** | **Data extraction—details** | **Location (PG, URL etc)** | |  | **‘Reasons for rating’: eg, ‘not reported or not clearly described’** |
|  |  |  | **Primary paper** | **Other^*^** | **YES: 1**  **NO: 0** |  |
| 1 | Detailed description of the type of exercise equipment | Whole-body vibration machine (TT2590X7, TurboSonic Co., Seoul, Korea). |  |  | 1 |  |
| 2 | Detailed description of the qualifications, expertise and/or training | The participants were evaluated before and 1–2 days after training for 2 weeks by three well-trained physical therapists, who were not informed on the participants and the purpose of this study. | Page 2 |  | 1 |  |
| 3 | Describe whether exercises are performed individually or in a group | NA |  |  | 0 |  |
| 4 | Describe whether exercises are supervised or unsupervised; how they are delivered | The exercises were performed under the supervision of a physiotherapist with over 5 years of experience. | Page 3 |  | 1 |  |
| 5 | Detailed description of how adherence to exercise is measured and reported | NA |  |  | 0 |  |
| 6 | Detailed description of motivation strategies | NA |  |  | 0 |  |
| 7a | Detailed description of the decision rule(s) for determining exercise progression | NA |  |  | 0 |  |
| 7b | Detailed description of how the exercise program was progressed | NA |  |  | 0 |  |
| 8 | Detailed description of each exercise to enable replication | During squatting, patients were instructed to place their feet side-by-side on the vibration platform and hold onto the handrail with their knees bent 30–45 degrees. When holding a bridge, patients were instructed to place both feet on the vibration platform and lift their hips, with their arms resting next to their trunk. Single bridge involved lifting one foot while holding a bridge. Bridge and knee flex involved holding a bridge with both knees bent 90 degrees. During side bridge, patients were instructed to lie on their side and support their weight with their forearms, knees, and feet, and to place their forearms on a vibration platform. When planking, patients were asked to be on their hands and knees with their hips and knees bent 90 degrees and both forearms placed on the vibration platform. The control group performed the same exercise for the same time without whole body vibration. Spine alignment was to be maintained in a neutral position at all times. | Page 3 |  | 1 |  |
| 9 | Detailed description of any home programme component | NA |  |  | 0 |  |
| 10 | Describe whether there are any non-exercise components | NA |  |  | 0 |  |
| 11 | Describe the type and number of adverse events that occur during exercise | No such incidents have occurred. |  |  | 1 |  |
| 12 | Describe the setting in which the exercises are performed | NA |  |  | 0 |  |
| 13 | Detailed description of the exercise intervention | The subjects, who were part of the Vibration (TT2590X7, TurboSonic Co., Seoul, Korea) group, performed six exercises (squat, bridge, single bridge, bridge and knee flex, side bridge, and plank) for 15 min on a Whole-body vibration machine (TT2590X7, TurboSonic Co., Seoul, Korea). There was a 5 min warm-up and cool down before and after exercise. Each exercise was performed for 60 s (single bridge, bridge and knee flex, plank) or 90 s (squat, bridge, and side bridge) for two sets, followed by a 30 s break after each set. | Page 3 |  | 1 |  |
| 14a | Describe whether the exercises are generic (one size fits all) or tailored | Referring to the previous study that suggested that 15 Hz is the most effective in promoting muscle activity of the core muscle during trunk stabilization exercise, we set the frequency to 15 Hz and the amplitude to 2 mm during all exercise | Page 3 |  | 1 |  |
| 14b | Detailed description of how exercises are tailored to the individual | NA |  |  | 0 |  |
| 15 | Describe the decision rule for determining the starting level | NA |  |  | 0 |  |
| 16a | Describe how adherence or fidelity is assessed/measured | NA |  |  | 0 |  |
| 16b | Describe the extent to which the intervention was delivered as planned | NA |  |  | 0 |  |
| Total score |  |  |  |  | 7 |  |
| *eg, protocol paper, published reference | | | | | | |

| **Table 2 Proforma CERT assessment form** | | | | | | |
| --- | --- | --- | --- | --- | --- | --- |
| **Author and year： Evans et al. 2018** | | | | | | |
| **Title: Spinal manipulation and exercise for low back pain in adolescents: a randomized trial** | | | | | | |
| **Journal: Pain** | | | | | | |
| **Study location: USA** | | | | | | |
| **Reviewer and date** | | | | | | |
| **Item** | **Description** | **Data extraction—details** | **Location (PG, URL etc)** | | **YES: 1**  **NO: 0** | **‘Reasons for rating’: eg, ‘not reported or not clearly described’** |
|  |  |  | **Primary paper** | **Other^*^** |  |  |
| 1 | Detailed description of the type of exercise equipment |  |  |  | 0 |  |
| 2 | Detailed description of the qualifications, expertise and/or training | All study personnel were trained and certified to implement study protocols in an effort to ensure standardization within and across sites. | Page: 4, |  | 1 |  |
| 3 | Describe whether exercises are performed individually or in a group | They were provided instructions to perform the same exercises at home and to engage in 20–40 minutes of aerobic activity twice per week. | Page: 4, |  | 1 |  |
| 4 | Describe whether exercises are supervised or unsupervised; how they are delivered | The ET program included self-care education, supervised exercise and instructions for home exercise. | Page: 4, |  | 0 |  |
| 5 | Detailed description of how adherence to exercise is measured and reported | Participant demographic and clinical characteristics were collected during the baseline visits via a comprehensive health history and physical examination and self-report questionnaires. | Page: 4, |  | 1 |  |
| 6 | Detailed description of motivation strategies | Selfcare education included patient-centered goal setting and emphasis on the importance of movement and activity, pain management, and spinal posture awareness with basic activities of daily living (e.g., sitting, getting out of bed and using a backpack). Participants were also provided printed instructions and photos for each exercise, along with a modified Back in Actionbook. | Page: 4, |  | 1 |  |
| 7a | Detailed description of the decision rule(s) for determining exercise progression | NA |  |  | 0 |  |
| 7b | Detailed description of how the exercise program was progressed | NA |  |  | 0 |  |
| 8 | Detailed description of each exercise to enable replication | Participants were also provided printed instructions and photos for each exercise, along with a modified Backin Actionbook | Page: 4, |  | 1 |  |
| 9 | Detailed description of any home programme component | They were provided instructions to perform the same exercises at home and to engage in 20–40 minutes of aerobic activity twice per week. | Page: 4, |  | 1 |  |
| 10 | Describe whether there are any non-exercise components | The ET program included self-care education, supervised exercise and instructions for home exercise；Participants were also provided printed instructions and photos for each exercise, along with a modified Backin Actionbook | Page: 4, |  | 1 |  |
| 11 | Describe the type and number of adverse events that occur during exercise | During the 12-week intervention, 5 participants reported visits to other healthcare providers for their LBP: 2 from the SMT+ ET group and 3 from ET alone. Between weeks 12 and 52, a total of 50 individuals sought additional healthcare: 21 in SMT+ ET (15 sought additional SMT) and 29 in ET alone (18 sought additional SMT). | Page: 7, |  | 1 |  |
| 12 | Describe the setting in which the exercises are performed | Some of the exercises mention the environment, such as home-based workouts. | Page: 4, |  | 1 |  |
| 13 | Detailed description of the exercise intervention | The ET program included self-care education, supervised exercise, and instructions for home exercise. Participants attended 816, 45-minute sessions with an exercise therapist or licensed chiropractor, no more than 2 times per week. Treatment dose was determined based on patients’ abilities and needs； he goal of the combined SMT+ ET program was to enhance patients’ ability to exercise by providing treatment to the lumbar vertebral or sacroiliac joints in an effort to increase mobility and decrease pain | Page: 4, |  | 1 |  |
| 14a | Describe whether the exercises are generic (one size fits all) or tailored | The treatment dose was determined based on the patients’ abilities and needs. Self-care education included patient-centered goal setting and emphasis on the importance of movement and activity, pain management, and spinal posture awareness with basic activities of daily living (e.g., sitting, getting out of bed, and using a backpack)； SMT dose, spinal levels treated, and technique were individualized to the patient based on the patient’s prognosis, tolerance, and needs. | Page: 4, |  | 1 |  |
| 14b | Detailed description of how exercises are tailored to the individual | The treatment dose was determined based on the patients’ abilities and needs. Self-care education included patient-centered goal setting and emphasis on the importance of movement and activity, pain management, and spinal posture awareness with basic activities of daily living (e.g., sitting, getting out of bed, and using a backpack). | Page: 4, |  | 1 |  |
| 15 | Describe the decision rule for determining the starting level | Participants began with exercises appropriate for their fitness level and progressed in difficulty by changing body position and/or labile surface (i.e. gym ball). | Page: 4, |  | 0 |  |
| 16a | Describe how adherence or fidelity is assessed/measured | Overall, 91% of study participants attended their prescribed treatment visits: 96% in the SMT+ ET group and 87% in the ET alone group. The mean number of ET visits was 10.8 (SD=1.8; median=11.0) in the SMT+ ET group and 9.8 (SD=3.0; median=11.0) in the ET alone group. The mean number of SMT visits was 10.1 (SD=1.9; median=10) in the SMT+ ET group. | Page: 7, |  | 1 |  |
| 16b | Describe the extent to which the intervention was delivered as planned | Overall, 91% of study participants attended their prescribed treatment visits: 96% in the SMT+ ET group and 87% in the ET alone group. The mean number of ET visits was 10.8 (SD=1.8; median=11.0) in the SMT+ ET group and 9.8 (SD=3.0; median=11.0) in the ET alone group. The mean number of SMT visits was 10.1 (SD=1.9; median=10) in the SMT+ ET group. | Page: 7, |  | 1 |  |
| Total score |  |  |  |  | 14 |  |
| *eg, protocol paper, published reference | | | | | | |

| **Table 3 Proforma CERT assessment form** | | | | | | |
| --- | --- | --- | --- | --- | --- | --- |
| **Author and year: Ahlqwist et al. (2008)** | | | | | | |
| **Title: Physical Therapy Treatment of Back Complaints on Children and Adolescents** | | | | | | |
| **Journal: Spine** | | | | | | |
| **Study location: Sweden** | | | | | | |
| **Reviewer and date** | | | | | | |
| **Item** | **Description** | **Data extraction—details** | **Location (PG, URL etc)** | |  | **‘Reasons for rating’: eg, ‘not reported or not clearly described’** |
|  |  |  | **Primary paper** | **Other^*^** | **YES: 1**  **NO: 0** |  |
| 1 | Detailed description of the type of exercise equipment | NA |  |  | 0 |  |
| 2 | Detailed description of the qualifications, expertise and/or training | Children were called for a first appointment and randomly divided between two licensed physiotherapists who specialized in Orthopedic Manual Therapy and Mechanical Diagnostic Therapy. | Page: 2 |  | 1 |  |
| 3 | Describe whether exercises are performed individually or in a group | NA |  |  | 0 |  |
| 4 | Describe whether exercises are supervised or unsupervised; how they are delivered | The physiotherapist contacted the child by telephone halfway through the training program. | Page: 3 |  | 1 |  |
| 5 | Detailed description of how adherence to exercise is measured and reported | NA |  |  | 0 |  |
| 6 | Detailed description of motivation strategies | NA |  |  | 0 |  |
| 7a | Detailed description of the decision rule(s) for determining exercise progression |  |  |  | 1 |  |
| 7b | Detailed description of how the exercise program was progressed | Resistance was gradually increased. The prescribed exercise dose was 15 repetitions/exercise at 60% of 1 RM. | Page: 3 |  | 1 |  |
| 8 | Detailed description of each exercise to enable replication | NA |  |  | 0 |  |
| 9 | Detailed description of any home programme component | Both groups exercised according to a standardized home exercise program. |  |  | 1 |  |
| 10 | Describe whether there are any non-exercise components | Group 1: Individualized physical therapy and exercise and self-training according to a standardized back exercise program and back education. Group 2: Self-training according to a standardized back exercise program with follow-up and back education. |  |  | 1 |  |
| 11 | Describe the type and number of adverse events that occur during exercise | NA |  |  | 0 |  |
| 12 | Describe the setting in which the exercises are performed | NA |  |  | 0 |  |
| 13 | Detailed description of the exercise intervention | The differences in treatment were that Group 1 was given individualized treatment and training under the supervision of the treating physical therapist. Treatment was given once a week for 12 weeks at the physical therapy clinic. The program included exercises for conditioning, mobility, strength, and coordination. Resistance was provided either using the body weight for resistance or with circuit training equipment.29 Children were treated as needed with methods such as Orthopedic Manual Therapy and Mechanical Diagnostic Therapy. Both active and passive movements were used to improve back and lower extremity mobility. Both general and specific exercises were used to improve the general strength and endurance of regional stabilizing abdominal and back muscles.30 Resistance was gradually increased. The prescribed exercise dose was 15 repetitions/exercise at 60% of 1 RM.11 Condition was improved using a stationary bicycle and treadmill for at least 20 minutes. Group 2 worked on conditioning through brisk walks, jogging, bicycling, or swimming 3 times a week for at least 20 minutes. The program was followed up at the clinic after 1 week. The physiotherapist contacted the child by telephone halfway through the training program. | Page: 3 |  | 1 |  |
| 14a | Describe whether the exercises are generic (one size fits all) or tailored | Both groups exercised according to a standardized home exercise program. | Page: 3 |  | 1 |  |
| 14b | Detailed description of how exercises are tailored to the individual | The differences in treatment were that Group 1 was given individualized treatment and training under the supervision of the treating physical therapist.; Resistance was gradually increased. The prescribed exercise dose was repetitions/exercise at 60% of 1 RM. | Page:3 |  | 1 |  |
| 15 | Describe the decision rule for determining the starting level | The exercises were done using the body weight for resistance with 2 sets of 10 repetitions each. | Page:3 |  | 1 |  |
| 16a | Describe how adherence or fidelity is assessed/measured | NA |  |  | 0 |  |
| 16b | Describe the extent to which the intervention was delivered as planned | NA |  |  | 0 |  |
| Total score |  |  |  |  | 10 |  |
| *eg, protocol paper, published reference | | | | | | |

| **Table 4 Proforma CERT assessment form** | | | | | | |
| --- | --- | --- | --- | --- | --- | --- |
| **Author and year: Fanucchi et al. (2009)** | | | | | | |
| **Title: Exercise reduces the intensity and prevalence of low back pain in 12–13 year old children: a randomised trial** | | | | | | |
| **Journal: Australian Journal of Physiotherapy** | | | | | | |
| **Study location: South Africa** | | | | | | |
| **Reviewer and date:** | | | | | | |
| **Item** | **Description** | **Data extraction—details** | **Location (PG, URL etc)** | |  | **‘Reasons for rating’: eg, ‘not reported or not clearly described’** |
|  |  |  | **Primary paper** | **Other^*^** | **YES: 1**  **NO: 0** |  |
| 1 | Detailed description of the type of exercise equipment | NA |  |  | 0 |  |
| 2 | Detailed description of the qualifications, expertise and/or training | A qualified, registered physiotherapist with five years’ experience in exercise rehabilitation, supervised all three exercise classes each week. In addition, the therapist received further training in the specific exercise program for this study. | Page: 5 |  | 1 |  |
| 3 | Describe whether exercises are performed individually or in a group | The experimental group participated in an 8-week exercise program during school hours (see Appendix 1 on the eAddenda for detailed information of the intervention). | Page: 2 |  | 1 |  |
| 4 | Describe whether exercises are supervised or unsupervised; how they are delivered | A qualified, registered physiotherapist with five years’ experience in exercise rehabilitation, supervised all three exercise classes each week. | Page: 5 |  | 0 |  |
| 5 | Detailed description of how adherence to exercise is measured and reported | NA |  |  | 0 |  |
| 6 | Detailed description of motivation strategies | NA |  |  | 0 |  |
| 7a | Detailed description of the decision rule(s) for determining exercise progression | NA |  |  | 0 |  |
| 7b | Detailed description of how the exercise program was progressed | NA |  |  | 0 |  |
| 8 | Detailed description of each exercise to enable replication | The experimental group participated in an 8-week exercise program during school hours (see Appendix 1 on the eAddenda for detailed information of the intervention). | Page: 2 |  | 1 |  |
| 9 | Detailed description of any home programme component | The control group received no intervention, ie, they did not attend the exercise classes nor did they take part in the home exercise program. | Page: 2 |  | 1 |  |
| 10 | Describe whether there are any non-exercise components | The exercise program consisted of eight classes of 40–45 minutes duration. The program was initiated with a 10–15 minute educational session. | Page: 2 |  | 1 |  |
| 11 | Describe the type and number of adverse events that occur during exercise | One child from the control group was lost to the study as she changed schools, and one child in the experimental group was excluded from the analysis as he sustained a serious back injury one week prior to the Month 6 measurement. | Page: 5 |  | 1 |  |
| 12 | Describe the setting in which the exercises are performed | The experimental group participated in an 8-week exercise program during school hours | Page: 2 |  | 1 |  |
| 13 | Detailed description of the exercise intervention | The exercise program consisted of eight classes of 40–45 minutes duration. The program was initiated with a 10–15 minute educational session. A physiotherapist discussed the importance of the exercises which the children would be doing and how the exercises related to their low back pain. A simplified explanation of the core musculature, correct posture, and spinal alignment was also included. The experimental group also received a weekly home exercise program which included exercises that had been taught in the class; The control group received no intervention, ie, they did not attend the exercise classes nor did they take part in the home exercise program. Both groups continued with their normal physical education classes, sports, and physical activity. | Page: 2 |  | 1 |  |
| 14a | Describe whether the exercises are generic (one size fits all) or tailored | NA |  |  | 0 |  |
| 14b | Detailed description of how exercises are tailored to the individual | NA |  |  | 0 |  |
| 15 | Describe the decision rule for determining the starting level | NA |  |  | 0 |  |
| 16a | Describe how adherence or fidelity is assessed/measured | The majority of the children in the experimental group attended all of the eight exercise classes. Only 5 (13%) of the children missed more than one class. One-third of the children reported that they did the home exercise program regularly (three or more times a week), whilst 20% of the children reported minimal adherence to the program (less than twice a week). Only one child did not do any of the home exercises. The majority of children indicated that they enjoyed the exercise class (85%), felt that the exercises helped to make them feel better (97%) and to make their backs stronger (81%). | Page: 5 |  | 1 |  |
| 16b | Describe the extent to which the intervention was delivered as planned | One child from the control group was lost to the study as she changed schools, and one child in the experimental group was excluded from the analysis as he sustained a serious back injury one week prior to the Month 6 measurement. | Page: 5 |  | 1 |  |
| Total score |  |  |  |  | 10 |  |
| *eg, protocol paper, published reference | | | | | | |

| **Table 5 Proforma CERT assessment form** | | | | | | |
| --- | --- | --- | --- | --- | --- | --- |
| **Author and year: Jones et al. (2007)** | | | | | | |
| **Title: The Efficacy of Exercise as an Intervention to Treat Recurrent Nonspecific Low Back Pain in Adolescents** | | | | | | |
| **Journal: Pediatric Exercise Science** | | | | | | |
| **Study location: UK** | | | | | | |
| **Reviewer and date** | | | | | | |
| **Item** | **Description** | **Data extraction—details** | **Location (PG, URL etc)** | |  | **‘Reasons for rating’: eg, ‘not reported or not clearly described’** |
|  |  |  | **Primary paper** | **Other^*^** | **YES: 1**  **NO: 0** |  |
| 1 | Detailed description of the type of exercise equipment | NA |  |  | 0 |  |
| 2 | Detailed description of the qualifications, expertise and/or training | NA |  |  | 0 |  |
| 3 | Describe whether exercises are performed individually or in a group | NA |  |  | 0 |  |
| 4 | Describe whether exercises are supervised or unsupervised; how they are delivered | NA |  |  | 0 |  |
| 5 | Detailed description of how adherence to exercise is measured and reported | Over two thirds of the participants in both groups had been prevented from participation in physical activity because of the NSLBP, and in just over one half of these cases this absence was on a regular basis. One third of participants in both groups had been absent from school because of NSLBP, although in most cases (85%) this was only on one occasion. | Page: 4 |  | 1 |  |
| 6 | Detailed description of motivation strategies | NA |  |  | 0 |  |
| 7a | Detailed description of the decision rule(s) for determining exercise progression | NA |  |  | 0 |  |
| 7b | Detailed description of how the exercise program was progressed | NA |  |  | 0 |  |
| 8 | Detailed description of each exercise to enable replication | NA |  |  | 0 |  |
| 9 | Detailed description of any home programme component | Home-based exercise tasks of pain-relieving level were also encouraged. | Page: 5 |  | 0 |  |
| 10 | Describe whether there are any non-exercise components | Participants took part in two structured group sessions per week over an 8-week period, and each session lasted approximately 30 min. | Page: 4 |  | 1 |  |
| 11 | Describe the type and number of adverse events that occur during exercise | From the 62 consenting participants, 54 completed the study; 2 participants from each group dropped out, and their partners were also removed from analysis; All participants who were included in the experimental group attended at least 12 of the 16 exercise sessions. No participants were prevented from continuing any of the exercise sessions as a result of NSLBP; it might have been a cause for absence from a specific session, although cause of absences was not determined. Overall, there was an 88% compliance rate to the exercise program. | Page: 2; Page: 3 |  | 1 |  |
| 12 | Describe the setting in which the exercises are performed | The program was completed in a school-based setting. | Page: 5 |  | 1 |  |
| 13 | Detailed description of the exercise intervention | The exercise rehabilitation program consisted of a combination of strength, flexibility, and aerobic exercises, in line with recommendations by McGill (20). Participants took part in two structured group sessions per week over an 8-week period, and each session lasted approximately 30 min. The program was a timecontingent progressive program, including pain relieving, reconditioning, and progressive exercises (20). | Page: 4 |  | 1 |  |
| 14a | Describe whether the exercises are generic (one size fits all) or tailored | All participants progressed through the exercise program at the same rate. The exercise program was fully standardized to include a specific number of individual exercises and repetitions and followed a prescribed exercise schedule (available on request). | Page: 5 |  | 1 |  |
| 14b | Detailed description of how exercises are tailored to the individual | NA |  |  | 0 |  |
| 15 | Describe the decision rule for determining the starting level | NA |  |  | 0 |  |
| 16a | Describe how adherence or fidelity is assessed/measured | Participants were interviewed following a standardized schedule to confirm the characteristics of their NSLBP; Over two thirds of the participants in both groups had been prevented from participation in physical activity because of the NSLBP, and in just over one half of these cases this absence was on a regular basis. One third of participants in both groups had been absent from school because of NSLBP, although in most cases (85%) this was only on one occasion. | Page: 4 |  | 1 |  |
| 16b | Describe the extent to which the intervention was delivered as planned | From the 62 consenting participants, 54 completed the study; 2 participants from each group dropped out, and their partners were also removed from analysis. | Page: 2 |  | 1 |  |
| Total score |  |  |  |  | 8 |  |
| *eg, protocol paper, published reference | | | | | | |
